# A Multiple Interaction Analysis Reveals ADRB3 as a Potential Candidate for Gallbladder Cancer Predisposition via a Complex Interaction with Other Candidate Gene Variations

**DOI:** 10.3390/ijms161226077

**Published:** 2015-11-25

**Authors:** Rajani Rai, Jong Joo Kim, Sanjeev Misra, Ashok Kumar, Balraj Mittal

**Affiliations:** 1 School of Biotechnology, Yeungnam University, Gyeongsan, Gyeongbuk 712-749, Korea; kimjj@ynu.ac.kr; 2 Department of Genetics, Sanjay Gandhi Post Graduate Institute of Medical Sciences (SGPGIMS), Lucknow-226014, India; 3 Department of Surgical Oncology, King George’s Medical University (KGMU), Lucknow-226003, India; misralko@gmail.com; 4 Department of Surgical Gastroenterology, Sanjay Gandhi Post Graduate Institute of Medical Sciences (SGPGIMS), Lucknow-226014, India; dr_ashokgupta@yahoo.com

**Keywords:** genetic susceptibility, polymorphism, gallbladder cancer (GBC), Multifactor-Dimensionality Reduction (MDR), Classification and Regression Tree Analysis (CRT)

## Abstract

Gallbladder cancer is the most common and a highly aggressive biliary tract malignancy with a dismal outcome. The pathogenesis of the disease is multifactorial, comprising the combined effect of multiple genetic variations of mild consequence along with numerous dietary and environmental risk factors. Previously, we demonstrated the association of several candidate gene variations with GBC risk. In this study, we aimed to identify the combination of gene variants and their possible interactions contributing towards genetic susceptibility of GBC. Here, we performed Multifactor-Dimensionality Reduction (MDR) and Classification and Regression Tree Analysis (CRT) to investigate the gene–gene interactions and the combined effect of 14 SNPs in nine genes (*DR4* (rs20576, rs6557634); *FAS* (rs2234767); *FASL* (rs763110); *DCC* (rs2229080, rs4078288, rs7504990, rs714); *PSCA* (rs2294008, rs2978974); *ADRA2A* (rs1801253); *ADRB1* (rs1800544); *ADRB3* (rs4994); *CYP17* (rs2486758)) involved in various signaling pathways. Genotyping was accomplished by PCR-RFLP or Taqman allelic discrimination assays. SPSS software version 16.0 and MDR software version 2.0 were used for all the statistical analysis. Single locus investigation demonstrated significant association of *DR4* (rs20576, rs6557634), *DCC* (rs714, rs2229080, rs4078288) and *ADRB3* (rs4994) polymorphisms with GBC risk. MDR analysis revealed *ADRB3* (rs4994) to be crucial candidate in GBC susceptibility that may act either alone (*p* < 0.0001, CVC = 10/10) or in combination with *DCC* (rs714 and rs2229080, *p* < 0.0001, CVC = 9/10). Our CRT results are in agreement with the above findings. Further, *in-silico* results of studied SNPs advocated their role in splicing, transcriptional and/or protein coding regulation. Overall, our result suggested complex interactions amongst the studied SNPs and *ADRB3* rs4994 as candidate influencing GBC susceptibility.

## 1. Introduction

Gallbladder cancer (GBC) is infrequent, however, it is very aggressive, and also the most common biliary tract cancer worldwide with marked geographical, racial and gender-specific orientations [1,2]. The etiology of GBC is multifactorial with gallstone and chronic inflammation as the root of disease [3,4]. Due to the absence of specific symptoms and late presentation, more than 90% of GBC patients are diagnosed at an advanced stage with little treatment alternatives [5]. Owing to unsatisfactory treatment options, the five-year survival rate is less than 5% and neither chemotherapy nor radiotherapy have been shown to improve the overall quality of life [6]. Further, despite recent advancements, the molecular basis of GBC is poorly understood and it still remains a diagnostic and therapeutic challenge for clinicians [7]. Thus, there is always a need to develop novel biomarker for its early diagnosis, and to enhance our understanding of inter-individual variability in vulnerability of GBC.

Previously, we have examined the role of various candidate gene variations in GBC patients from North India. These variants are part of genes involved in various signaling pathways, including apoptosis, cell survival, cell–cell interaction, estrogen metabolism, *etc.* [8,9,10,11,12]. However, being low penetrance genetic variations, their individual contribution towards GBC is very small and single SNPs cannot exactly account for GBC susceptibility. Further, carcinogenesis is a highly intricate process and polygenic in nature involving multiple gene variations of mild consequence [13]. In addition, gene–gene and gene–environment interactions are believed to be major players in the pathogenesis of GBC and can modulate individual’s susceptibility to cancer [14]. Hence, we have extended our earlier work by simultaneously exploring 14 polymorphisms in nine genes (*DR4*: A>C (rs20576), G>A (rs6557634); *FAS*-1377G>A (rs2234767); *FASL*-844T>C (rs763110); *DCC*:C >G (rs2229080), A>G (rs4078288), C>T (rs7504990), A>G (rs714); *PSCA*:C>T (rs2294008), G>A (rs2978974); *ADRA2A*-1291C>G (rs1801253); *ADRB1* 1165C>G (rs1800544); *ADRB3* 190T>C (rs4994); *CYP17* T>C (rs2486758)) by using Multifactor-Dimensionality Reduction (MDR) method and classification and regression trees (CRT) to determine possible higher order gene–gene interactions and accomplish a comprehensive appraisal of GBC risk. These are a non-parametric, genetic model-free methodologies [15] having advantage to identify association in studies having small sample sizes and low penetrance of candidate SNPs as compared to previous traditional methods such as logistic regression [16,17].

## 2. Results

The demographic profile of GBC patients and controls are displayed in Table 1. The mean age of 400 GBC cases and 246 controls were 52.65 ± 10.45 and 47.75 ± 10.65 years, respectively. Most of the GBC patients (~95%) were in stage III and stage IV of cancer.

**Table 1:** Characteristic of the Study Subjects.

Variables	Cases N (%)	Controls N (%)
Whole Subjects	400 (100)	246 (100)
Female	278 (69.5)	163 (66.3)
Male	122 (30.5)	83 (33.7)
Age ± SD	52.65 ± 10.45	47.75 ± 10.65
Stages		
0, I	None	NA
II	21 (5.25)	
III	199 (49.75)	
IV	180 (45.0)	
Gallstone present	200 (50.0)	None
Gallstone absent	200 (50.0)	246 (100)
Tobacco		
No	273 (68.9)	NA
Yes	123 (31.1)	

NA: not available.

### 2.1. Single Locus Analysis

Table 2 represents the association of all the studied SNPs with GBC risk. The heterozygous genotypes of *DR4* (rs20576, rs6557634), and variant genotype of *DCC* rs4078288 was found to confer significantly increased risk of GBC (adjusted OR > 1; *p* < 0.05). Further, both hetero- and variant-genotypes of *DCC* rs714 and ADRB3 rs4994 were associated with the increased susceptibility of GBC, whereas genotype containing at least one variant allele of *DCC* rs2229080 was found to confer protection against GBC risk.

**Table 2:** Single locus analysis of SNPs investigated.

Pathway	Gene	SNP	MAF_controls_	MAF_cases_	OR_het_^a^	OR_hom_^a^
Death receptor	*Dr4*	rs20576	8	14	1.82 (1.18–2.83)	3.27 (0.93–11.51)
		rs6557634	27	33	1.61 (1.06–2.44)	2.05 (0.90–4.70)
	*FAS*	rs763110	39	41	0.94 (0.66–1.33)	1.26 (0.78–2.02)
	*FASL*	rs2234767	20	22	0.99 (0.71–1.38)	1.66 (0.70–4.12)
Tumor suppressor	*DCC*	rs714	37	45	1.84 (1.29–2.63)	1.72 (1.08–2.74)
		rs2229080	32	24	0.64 (0.46–0.89)	0.32 (0.15–0.68)
		rs7504990	32	31	1.01 (0.72–1.40)	0.92 (0.51–1.65)
		rs4078288	34	39	0.98 (0.69–1.39)	1.58 (1.01–2.49)
Prostate stem cell antigen	*PSCA*	rs2978974	32	30	0.91 (0.65–1.27)	0.86 (0.50–1.48)
		rs2294008	42	46	1.4 (0.97–2.02)	1.25 (0.77–2.04)
Adrenergic pathway	*ADRa2a*	rs1800544	45	49	1.35 (0.92–1.97)	1.41 (0.87–2.29)
	*ADRB3*	rs4994	10	21	2.58 (1.76–3.78)	10.61 (1.38–81.92)
	*ADRB1*	rs1801253	22	25	1.32 (0.95–1.84)	1.12 (0.46–2.78)
Estrogen metabolism pathway	*CYP17*	rs2486758	26	27	1.04 (0.74–1.45)	1.11 (0.59–2.09)

Significant values are denoted as bold. ^a^ Adjusted for age and gender in logistic regression model; OR_het_: odds ratio of heterozygote *vs.* common homozygote genotypes; OR_hom_: odds ratio of homozygote *vs.* common homozygote genotypes, MAF: Minor allele frequency.

### 2.2. Multifactor Dimensionality Reduction (MDR)

Our MDR analysis demonstrated *ADRB3*_rs4994_ polymorphism (testing accuracy = 0.6003, CVC = 10/10, *p* < 0.0001) as the one-factor model for envisaging the GBC risk. *DCC*_rs2229080_, *ADRB3*_rs4994_ constitutes the two-factor model with testing accuracy of 0.5658 but CVC = 6/10 (*p* < 0.0001). The three-factor model, comprising *DCC*_rs714_, DCC_rs2229080_, and *ADRB3*_rs4994_ SNPs had the improved testing accuracy of 0.5913 and the CVC of 9/10 (*p* ≤ 0.0001). Likewise, *DCC*_rs714_, *DCC*_rs2229080_, *PSCA*_rs2978974_, and *ADRB3*_rs4994_ polymorphisms represents the four-factor interaction model, having a testing accuracy of 0.5353 and CVC = 3/10 with *p* < 0.0001 (Table 3).

**Table 3:** Multifactor dimensionality reduction (MDR) analysis showing association of high-order interactions with GBC.

No. of Risk Factors	Best Interaction Model	Testing Accuracy	^#^ CVC	*X*² (*p*-Value)	OR (95% CI)
1	*ADRB3*_rs4994_	0.6003	10/10	28.5717 (*p* < 0.0001)	2.7507 (1.8841–4.0158)
2	*DCC*_rs2229080_, *ADRB3*_rs4994_	0.5658	6/10	32.5889 (*p* < 0.0001)	2.6238 (1.8762–3.6693)
3	*DCC*_rs714_, *DCC*_rs2229080_, *ADRB3*_rs4994_	0.5913	9/10	44.324 (*p* < 0.0001)	3.0155 (2.1684–4.1935)
4	*DCC*_rs714_, *DCC*_rs2229080_, *PSCA*_rs2978974_, *ADRB3*_rs4994_	0.5353	3/10	68.7203 (*p* < 0.0001)	4.0443 (2.8834–5.6726)

^#^ The model with maximum testing accuracy and maximum CVC cross was considered as the best model; CVC: cross-validation consistency.

### 2.3. Classification and Regression Tree Analysis (CRT)

**Figure 1:**
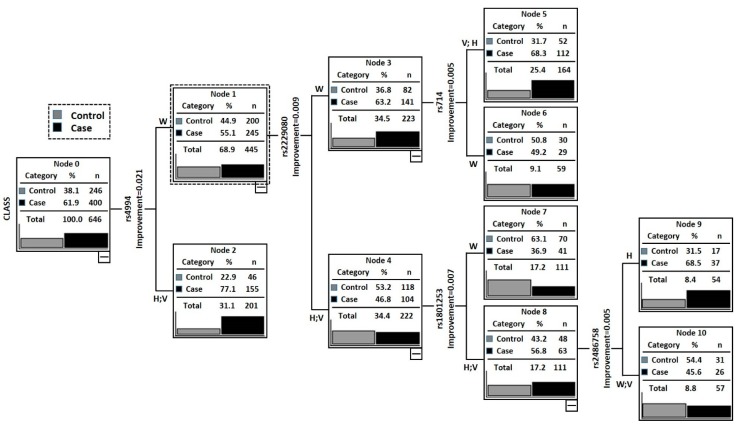
Classification and regression tree model for selected 14 SNPs and GBC risk. Terminal nodes at the end. W: Wild type genotype; V: Variant genotype; H: heterozygous.

Figure 1 depict the results of CRT analysis, containing all the studied SNPs. The tree comprised of total eleven nodes and six terminal nodes (node that has no child nodes) with *ADRB3*_rs4994_ polymorphism lying at the top of tree signifying it as the main contributing factor for GBC. Subjects with *ADRB3*_rs4994_ (W), *DCC*_rs2229080_ (H + V) and *ADRB1*_rs1801253_ (W) genotypes (Node 1) having the lowest case rate (36.94%) was taken as reference.

Table 4 summarizes the risk associated with all the terminal nodes compared with Node 1 (*ADRB3*_rs4994_ (W) + *DCC*_rs2229080_ (H + V) +*ADRB1*_rs1801253_ (W). Subjects having the *ADRB3*_rs4994_ (W) + *DCC*_rs2229080_ (W) + *DCC*_rs714_ (H + V) and *ADRB3*_rs4994_ (W) + *DCC*_rs2229080_ (H + V) + *ADRB1*_rs1801253_ (H + V) + *Cyp17*_rs2486758_ (H) genotypes were found to have a significantly increased GBC susceptibility (adjusted OR = 3.7; *p* = 0.0003 and OR = 3.7; *p* = 0.0001). Importantly, all the terminal nodes were comprised of *ADRB3* rs4994 and *DCC*_rs2229080_ polymorphism (Table 4).

**Table 4:** Risk estimate based on Classification and Regression Tree Analysis (CRT) terminal nodes.

Nodes	Genotype of Individuals in Each Node	Case	Control	Total	Case Rate (%)	*p*-Value	OR (95% CI) ^a^
Node 1	*ADRB3*_rs4994_ (W) + *DCC*_rs2229080_ (H + V) + *ADRB1*_rs1801253_ (W)	41	70	111	36.94	–	Reference
Node 2	*ADRB3*_rs4994_ (W) + *DCC*_rs2229080_ (H + V) + *ADRB1*_rs1801253_ (H + V) + *Cyp17*_rs2486758_ (W + V)	26	31	57	45.61	0.2836	1.43 (0.74–2.75)
Node 3	*ADRB3*_rs4994_ (W) + *DCC*_rs2229080_ (W) + *DCC*_rs714_ (W)	29	30	59	49.15	0.1290	1.65 (0.87–3.14)
Node 4	*ADRB3*_rs4994_ (W) + *DCC*_rs2229080_ (W) + *DCC*_rs714_ (H + V)	112	52	164	68.29	0.0003	3.66 (2.21–6.12)
Node 5	*ADRB3*_rs4994_ (W) + *DCC*_rs2229080_ (H + V) + *ADRB*1_rs1801253_ (H + V) + *Cyp17*_rs2486758_ (H)	37	17	54	68.52	0.0001	3.69 (1.86–7.50)

Case rate: Percentage of cancer patients among all individuals in each node (case/(case + control) × 100); ^a^ Adjusted for age and gender.

### 2.4. In-Silico Analysis

The *in-silico* analyses of all the studied SNPs are shown in Table 5. MDR and CRT analysis demonstrated *ADRB3*_rs4994_ is the key causative factor in gallbladder carcinogenesis. Our *in-silico* analysis also showed this SNP to alter protein coding, splicing and transcriptional regulation. *DCC*_rs2229080_ polymorphism was also shown to change the protein coding and splicing regulation. *DCC*_rs714_ and *PSCA*_rs2978974_ are intronic SNPs with unknown function.

**Table 5:** Bioinformatic Analysis.

SNPs	Result of F-SNP/FAST SNP			
–	FS Score	Functional Category	Prediction Tool	Prediction Result
*DR4*_rs20576_	0	Protein coding	Ensemble	Nonsynnymous
			Polyphen	Possible damaging
*DR4*_rs6557634_	0.284	Protein coding	Ensembl	Nonsynonymous
			Polyphen	Probably damaging
		Splicing regulation	ESE finder	Changed
			ESR Search	Changed
*FASL*_rs763110_	0.434	Protein coding	Ensembl	Frameshift-coding
		Transcriptional regulation	TF-Search	Changed
			Ensembl-TR	Regulatory region
*FAS*_rs2234767_	0	Protein coding	Ensembl	Nonsynnymous
*DCC*_rs2229080_	0.616	Protein coding	Polyphen	Probably damaging
			SNPeffect	Deleterious
			LS-SNP	Deleterious
				Missense (non-conservative) Medium–high (3,4)
		Splicing regulation	ESE finder	Changed
			ESR Search	Changed
			PESX	Changed
*DCC*_rs4078288_	NA	Intronic enhancer Very low–low (1–2)		
*DCC*_rs7504990_	NA	Intronic with no known function		
*DCC*_rs714_	NA	Intronic with no known function		
*Cyp17*_rs2486758_	0.176	Transcriptional regulation	TFSearch	Changed
*ADRA2A*_rs1800544_	0.065	Transcriptional regulation	Golden path	Exit
*ADRB3*_rs4994_	0.551	Protein coding	Ensembl	Nonsynonymous
			SIFT	Damaging
			SNPeffect	Deleterious
		Splicing regulation	ESE finder	Changed
			ESR Search	Changed
			PESX	Changed
		Transcriptional regulation	Golden path	Exit
*ADRB1*_rs1800544_	0.774	Protein coding	Ensembl	Nonsynonymous
*CYP17*_rs2486758_	0.176	Transcriptional regulation	TFsearch	Changed

## 3. Discussion

Recent advancement in molecular biology has suggested extensive interactions amongst various genes or risk alleles (in which effect of single gene variation is influenced by other genetic variation *i.e.*, gene–gene interaction) as the key factor modulating the disease susceptibility. Hence, in this study, we aimed to investigate the synergistic effect of various gene variations to modulate GBC susceptibility instead of their individual effect, by using MDR and CRT. MDR improves the identification of multilocus genotype combinations (higher order gene–gene interactions) predicting the disease vulnerability for common, complex and multifactorial diseases [15]. CRT analysis, which is based on recursive partitioning the data space and fitting a simple prediction model within each partition, is a powerful technique with significant potential and clinical utility [18]. It categorizes the study subjects according to various risk levels on the basis of the various gene polymorphisms [19]. Both MDR and CRT are widely used in large-scale association studies because of their capability to overcome sample size limitations and the curse of dimensionality as compared to case-control studies using logistic regression [16,17].

Our single locus analysis showed *ARDB3*_rs4994_ as the important factor enhancing the GBC risk. The MDR analysis also showed *ADRB3*_rs4994_ alone as the best candidate with highest testing accuracy and CVC. Further, the three-factor interaction model consisting of *DCC*_rs714_, *DCC*_rs2229080_, *ADRB3*_rs4994_ constitutes the second best SNPs model with testing accuracy of 0.5913 and CVC = 9/10 (*p* < 0.001). The result of CRT analysis further affirmed *ADRB3*_rs4994_ as the major risk factor for GBC advancement. In addition, it corroborated MDR result and showed a complex interaction amongst *ADRB3*_rs4994_, *DCC*_rs2229080_, *DCC*_rs714_ as well as *Cyp17*_rs2486758_ attributing increased susceptibility to GBC. These finding suggested some correlation among these genes or proteins.

*ADRB3*, a member of class of G-protein-coupled receptor family, is abundantly distributed in adipose tissue and regulate lipolysis and thermogenesis [20]. In addition, it has been localized to vascular and nonvascular smooth muscle of human gastrointestinal tract, as well as in gallbladder regulating the blood flow and motility in gastrointestinal tract and gallbladder [21,22]. *ADRB3* rs4994 is a missense variation substituting tryptophan with arginine at codon 64. This SNP has been shown to influence fat accumulation and been implicated in the etiology of obesity that may serve as the predisposing factor for GBC [23,24]. It was also shown to alter the susceptibility to colon cancer risk in obese subjects [25]. Moreover, it has been established to increase the risk of gallstone disease, (a precancerous lesion for GBC), suggesting it as a gene marker for increased risk for gallstone [26,27]. In our previous study, we showed that *ADRB3*_rs4994_ conferred increased risk of GBC both by gallstone-dependent and -independent mechanisms [10]. Here, our multi-analytical approaches further confirmed the association of this SNP, either alone or in combination, with GBC risk. On the contrary, a recent study failed to show the association of this SNP with pancreatic cancer [28] may be due to different pathology underlying different organs.

*DCC* (netrin-1), originally discover in colorectal cancer, is characterized as a candidate tumor suppressor gene that encodes the netrin 1 receptor, a member of the immunoglobulin superfamily of cell adhesion molecules [29]. When *DCC* is present and bound to netrin-1 receptor, it induces cell proliferation and migration, while in the absence of netrin-1, an intracellular domain of *DCC* is cleaved by a caspase inducing apoptosis in a caspase-9-dependent pathway [30]. In various human cancers, it has been shown to be frequently silenced or inactivated due to loss of heterozygosity at chromosome 18q21 region or epigenetic silencing [29,31]. Loss of *DCC* gene expression was shown to be an independent prognostic factor in AML [32], colorectal [33] and gastric cancer [34,35] patients. Several studies have demonstrated significant association of *DCC* polymorphism with colorectal, esophageal, and gastric cancer risk [36,37,38,39]. The deletions at 18q21 loci (containing *DCC* gene) is an important step in the progression of GBC [40]. A genome-wide association study (GWAS) also suggested *DCC* as a candidate gene conferring GBC predisposition in a Japanese population [41]. In our previous work, we found no effect of GWAS reported SNPs on GBC risk. On the contrary, we showed significant association of *DCC* rs714 and rs2229080 with GBC risk [12]. The rs714 has been shown to be associated with loss of heterozygosity (LOH) and decreased expression of *DCC* in various cancers [42,43]. Further, rs2229080, a missense variation replacing Arg to Gly at DCC codon 201, was reported to increase the risk of colorectal cancer [44] and neuroblastoma [45]. Moreover, this SNP was suggested to be a target of LOH and associated with loss of *DCC* protein expression indicating that the codon 201 polymorphism may interfere with the *DCC* transcription or transition [46].

*PSCA*, originally identified as a prostate cell surface specific marker, was also established to be overexpressed in several other human cancers and suggested to play a role in carcinogenesis by regulating the cell proliferation, adhesion, migration and survival [47]. High expression of *PSCA* is significantly associated with adverse prognostic features and cancer severity, including; differentiation, invasion, metastasis and decreased overall survival [48,49]. The expression and function of *PSCA* are tissue specific, *i.e.*, it acts like tumor suppressor gene (TSG) in some organ while as oncogene (OG) in others. In GBC, it was reported to be downregulated and act like TSG by modulating immunological characteristics of GBC cells [50,51,52]. However, a recent study has shown *PSCA* overexpression in GBC that is associated with invasive potential and prognosis of GBC [49]. Further, several GWAS and case control studies have demonstrated association of *PSCA* gene polymorphisms rs2294008 and rs2976392 with various cancers, though some controversies also existed [48,53,54,55,56]. The *PSCA*_rs2294008_, located in exon 1, was found to affect the transcriptional activity [57,58] and the missense allele of rs2294008 was shown to attenuate antitumor activities of *PSCA* in GBC and consequently it was suggested to be a potential risk for GBC development [51]. The rs2976392G>A positioned in intron 2 is in strong linkage disequilibrium with rs2294008C>T, and its function is unclear till yet [59]. In our previous study, we failed to find the association of *PSCA* polymorphism with GBC risk, but on gender stratification, Trs2294008-Grs2978974 haplotype was found to confer higher risk of GBC in females (FDR Pcorr = 0.021), while Trs2294008-Ars2978974 haplotype is associated with significantly decreased risk in males (FDR Pcorr = 0.013) suggesting gender specific effect of *PSCA* haplotypes on GBC susceptibility [11]. Here, we found this SNP to increase GBC risk only in combination with *DCC* and *ADRB3* SNPs, though the CVC is low (3/10, *p* < 0.0001).

Our *in-silico* investigation of *ADRB3*_rs4994_ and *DCC*_rs2229080_ showed alteration in protein coding, splicing regulation and transcriptional regulation. *CYP17*_rs2486758_ was also found to alter transcriptional regulation. Other associated SNPs (*DCC*_rs714_, *PSCA*_rs2978974_) are intronic, hence our *in silico* study did not show any effect of these SNPs. Though, intronic SNPs are reported to be important player in splicing regulation and may affect other SNP lying in linkage disequilibrium.

Smoking/tobacco usage may be an important issue affecting disease susceptibility. However, we did consider smoking data due to non-reliability of collecting such information from controls. In earlier studies, we had therefore carried out case only analysis for modulation of genetic susceptibility by tobacco usage. However, in the present study, tobacco related analysis has not been done due to limited data. Here, we carried out only MDR and CART analysis for higher order gene–gene analysis.

## 4. Materials and Methods

### 4.1. Ethics Statement and Study Population

The present study was approved by the ethical committee of Sanjay Gandhi Postgraduate Institute of Medical Sciences (SGPGIMS). (Approval number: IEC code- 2012-170-EMP-66, approval date: 10.01.2013) Written informed consent was collected from all participants involved in the study.

A total of 646 subjects, including 400 GBC patients and 246 healthy control subjects of North Indian Ethnicity were recruited in this study from the department of Surgical Oncology, KGMU and Department of Surgical Gastroenterology SGPGIMS, Lucknow. The inclusion–exclusion criteria for cases and controls, and staging of cancer were same as previously reported in our studies [8,9,10,11,12]. In general, controls were frequency-matched to cancer cases for age, gender, and ethnicity, and were free from any history of malignancy as well as gallstones. For GBC cases, only confirmed subject (by FNAC; fine needle aspirated cell cytology or histopathology) were included in the study, while those already receiving chemotherapy were excluded.

### 4.2. Selected SNPs and Genotyping

In the present study, we have included *DR4*:A>C (rs20576), G>A (rs6557634); *FAS*-1377G>A (rs2234767); *FASL*-844T>C (rs763110); *DCC*:C>G (rs2229080), A>G (rs4078288), C>T (rs7504990), A>G (rs714); *PSCA*:C>T (rs2294008), G>A (rs2978974); *ADRA2A*-1291C>G (rs1801253); *ADRB1* 1165C>G (rs1800544); *ADRB3* 190T>C (rs4994); and *CYP17* T>C (rs2486758) SNPs.

Salting out method was used to isolate genomic DNA from 5 mL peripheral blood leukocytes [60]. The genotyping was performed by the PCR restriction fragment length polymorphism and TaqMan^®^ allelic discrimination assays (Applied Biosystems 7500 Fast Real-Time PCR (Thermo Fisher Scientific, Walthan, MA, USA)) method, as described previously [8,9,10,11,12]. PCR mix without DNA sample was taken as negative control and the 10% of random samples were sequenced to confirm the results consistency.

## 5. Statistical Analysis

### 5.1. Single Locus Analysis

Mean with standard deviation (SD) and absolute value were used for continuous and categorical measures, respectively. The frequency distributions of SNPs genotype between cases and controls were compared by using the chi-square analysis or two-sided Fisher’s exact test. Unconditional multivariate logistic regression (LR) was used to assess the odds ratios (ORs) and 95% confidence intervals (CIs) to estimate the risk of gallbladder cancer with the polymorphisms. The ORs were adjusted for age and gender. All statistical analyses were performed using SPSS software version 16.0 (SPSS, Chicago, IL, USA) and a *p*-value of less than 0.05 was considered a statistically significant.

### 5.2. Multifactor Dimensionality Reduction (MDR)

The MDR analysis was carried out by onine MDR software version 2.0 [61] producing several genotype interaction models. Amongst them, the genotype combination having the highest testing accuracy and the cross-validation consistency (CVC) is taken as the best interaction model [62]. The combined effect of the variables was calculated using LR analysis and a *p*-value less than 0.05 was considered to be statistically significant.

### 5.3. Classification and Regression Tree Analysis (CRT)

The SPSS software (version 16.0) was used to accomplish the CRT analysis producing a decision tree. In CRT analysis, starting with the core node comprising of the total sample, each node is divided into two child nodes repetitively by recursive partitioning [19], thus creating a tree like structure. The risk of all genotypes sets was estimated by considering the node with low case rate was as the reference to calculate the ORs and 95% CIs.

### 5.4. In-Silico Analysis and Functional Prediction of SNPs

Various online prediction tools such as; FASTSNP, F-SNP [63,64,65,66] were used to predict the functional effects of all the studied SNPs.

## 6. Conclusions

In conclusion, we found *ADRB3* as the main SNPs associated with increased GBC susceptibility. In addition, we showed a complex interaction amongst *ADRB3, DCC, PSCA* and *CYP17* increasing GBC risk. Further, our results allow more precise definition of subjects with high or low risk for GBC. Viewing the functional consequence of these SNPs in cancer initiation and progression, it is of great importance to further look into the underlying mechanism of carcinogenesis at gene levels and their interactive pathway. Future studies exploring the panels of the risk allele for GBC susceptibility in a larger sample size may have important implications in GBC management.
